# Efficacy and safety of several common drugs in the treatment of acute respiratory distress syndrome: A systematic review and network meta-analysis

**DOI:** 10.1097/MD.0000000000040472

**Published:** 2024-11-22

**Authors:** Qing-Kuo Liu, Guo-Han Xiang, Wen-Li Liu, Jin-Yan Dong, Yu-Qi Wen, Hao Hao

**Affiliations:** a Shandong University of Traditional Chinese Medicine, Jinan, Shandong, China; b Intensive Care Unit, Affiliated Hospital of Shandong University of Traditional Chinese Medicine, Jinan, Shandong, China.

**Keywords:** acute respiratory distress syndrome, inhaled pulmonary vasodilators, mesenchymal stem cells, neuromuscular blockers

## Abstract

**Background:**

This study aimed to compare the effectiveness and safety of neuromuscular blockers, mesenchymal stem cells (MSC), and inhaled pulmonary vasodilators (IV) for acute respiratory distress syndrome through a network meta-analysis of randomized controlled trials (RCTs).

**Methods:**

We searched Chinese and English databases, including China National Knowledge Infrastructure, The Cochrane Library, PubMed, and EMbase, with no time restrictions. We conducted a network meta-analysis and reported the results according to the Preferred Reporting Items for Systematic Reviews and Meta-Analyses. We included 27 clinical RCTs, all of which were two-arm trials, totaling 3492 patients. We selected 28-day mortality as the primary outcome measure, whereas 90-day mortality, ventilator-free days, and oxygenation served as secondary outcome measures for analysis and comparison.

**Results:**

We selected 3 treatment modalities and evaluated their clinical trials in comparison with the standard control group. For the 28-day in-hospital mortality, we included 21 RCTs, involving 2789 patients. Compared to standard treatment, neuromuscular blockers were associated with reduced 28-day hospital mortality (odds ratios [OR] 0.52, 95% confidence intervals [CI] (0.31, 0.88)), while IV and MSC were not associated with reduced hospital mortality (OR 0.89, 95% CI (0.50, 1.55); OR 0.90, 95% CI (0.49, 1.66)). In terms of 90-day mortality, days free of mechanical ventilation, and improvement in oxygenation, there were no significant differences compared to standard treatment with neuromuscular blockers, MSC, and IV.

**Conclusion:**

Neuromuscular blockers significantly reduced the 28-day mortality rate in acute respiratory distress syndrome patients. However, in terms of 90-day mortality, ventilator-free days, oxygenation improvement, IV, MSC, and neuromuscular blockers did not significantly improve.

## 1. Introduction

Acute respiratory distress syndrome (ARDS) is a clinical syndrome characterized by the onset of illness within 1 week, radiographic evidence of bilateral lung infiltrates, non-cardiogenic pulmonary edema, and refractory hypoxemia.^[1]^ ARDS is a common condition in critically ill patients. According to a large clinical observational study,^[2]^ the incidence of ARDS in critically ill patients is 10.4%, accounting for 23.4% of patients requiring mechanical ventilation. Hospital mortality rates for mild, moderate, and severe ARDS were 34.9%, 40.3%, and 46.1%, respectively. During the global Coronavirus Disease 2019 (COVID-19), studies^[3]^ have shown that ARDS induced by viral pneumonia is one of the main causes of death among COVID-19 hospitalized patients. The incidence of ARDS in COVID-19 hospitalized patients is 33%, with an average mortality rate of 39%. The etiology of ARDS includes direct lung injury (bacterial or viral pneumonia, aspiration pneumonia, and lung contusion) and extrapulmonary factors (sepsis, severe trauma, major surgery, and massive transfusions). The pathogenesis of ARDS is not yet fully understood. However, it is generally believed that its main mechanism involves a cascade of amplified inflammatory responses. As for the treatment of ARDS, effective measures primarily include lung-protective ventilation, control of driving pressure, prone positioning, and extracorporeal membrane oxygenation.^[4]^

The 2023 “ESICM Acute Respiratory Distress Syndrome Guidelines: Definition, Phenotypes, and Respiratory Support Strategies”^[5]^ explicitly recommend the use of corticosteroids for ARDS patients. It has been found that corticosteroids can reduce the mortality associated with COVID-19-related acute hypoxemic respiratory failure^[6]^ and severe community-acquired pneumonia.^[7]^ Therefore, corticosteroids were not included as an intervention measure in this study. Additionally, the guidelines recommend that in the early use of neuromuscular blocking agents(NBA) in mechanically ventilated ARDS patients, compared to patients not receiving neuromuscular blocking agent treatment, neuromuscular blocking agents can reduce the mortality of patients with moderate to severe ARDS, but the results are inconsistent and biased towards individual studies. A recent systematic review and meta-analysis (SR/MA) on the impact of neuromuscular blocking agents on ARDS^[8]^ showed that neuromuscular blocking agents can safely reduce mortality, but there is no significant reduction in ventilator-free days and ICU time. Another SR/MA^[9]^ showed that the effect of NBA infusion on mortality depended on the strategy used in the control group. Compared to deep sedation, mortality is reduced; however, compared to light sedation, there is no effect on mortality. A 2020 SR/MA^[10]^ evaluating the impact of NBA on the treatment of ARDS produced inconsistent results regarding the impact on mortality, with early use of NBA reducing the 21 to 28 day mortality but not improving the 90-day mortality. In an SR/MA on mesenchymal stem cell (MSC) treatment for ARDS,^[11]^ mortality was reduced in both general ARDS and COVID-19-induced ARDS, and adverse events did not differ between the MSC and control groups. However, in another phase I and II randomized controlled trial (RCT) SR/MA,^[12]^ MSC did not significantly reduce the 28-day mortality, although there were trends of improvement in oxygenation index and biomarkers during treatment, they lacked statistical significance, possibly due to the small sample size. In an evaluation of the treatment of ARDS with inhaled prostanoids, an SR/MA^[13]^ showed that using inhaled prostanoids can improve oxygenation and lower pulmonary artery pressure, but this SR/MA included 70% retrospective studies and exhibited significant heterogeneity. A systematic review of inhaled nitric oxide intervention for ARDS^[14]^ indicated a significant improvement in the PaO_2_/FiO_2_ ratio at 24 hours (mean difference (95% confidence intervals [CI]) 15.91 (8.25–23.56)), but no significant improvement at 48 or 72 hours, with no reduction in mortality, and it may lead to kidney damage. In a SR/MA of inhaled pulmonary vasodilators (IV) in COVID-19 infection,^[15]^ IV improved oxygenation compared to standard treatment alone but had no benefit on mortality. This study included retrospective or cohort studies; thus, large randomized required to verify the results.

Most current studies only focus on comparing the efficacy of a single drug with that of a placebo, and there is controversy regarding the effectiveness and safety of these drugs. No clinical trials have simultaneously compared the clinical efficacy and safety of 3 or more drugs using the same evidence base. Therefore, this study aimed to use network meta-analysis methods to integrate clinical evidence related to both direct and indirect comparisons and to quantitatively analyze and rank the combined statistics of different drugs for the same evidence base for treating this condition. This will help in selecting the best treatment options among different Chinese patent medicines based on their efficacy and safety, thereby providing a reference and evidence-based medicine for optimizing drug selection in clinical practice.

## 2. Materials and methods

### 2.1. Registration

The study protocol was registered with the International Prospective Register of Systematic Reviews (PROSPERO) under registration number CRD42023495105.

### 2.2. Data source

We strictly adhered to the Preferred Reporting Items for Systematic Reviews and Meta-Analyses reporting guidelines.^[16,17]^ Using computer retrieval, clinical RCTs on the treatment of ARDS using neuromuscular blocking agents, MSC, and pulmonary vasodilators were selected as primary retrievals. The retrieval was conducted up to September 5, 2023, and included Chinese databases such as the China National Knowledge Infrastructure, as well as English databases including The Cochrane Library, PubMed, and EMbase. The English search strategy: [Respiratory Distress Syndrome or acute respiratory distress syndrome or Respiratory Distress Syndromes or Shock Lung or Human ARDS or ARDS or Pediatric Respiratory Distress Syndrome or acute respiratory dysfunction syndrome and Mesenchymal Stem Cells or Bone Marrow Mesenchymal Stem Cells or Bone Marrow Stromal Cells or Multipotent Bone Marrow Stromal Cell or Adipose-Derived Mesenchymal Stem Cells or Adipose Tissue-Derived Mesenchymal Stem Cell or Mesenchymal Stromal Cells or Mesenchymal Progenitor Cell or Multipotent Mesenchymal Stromal Cell or Progenitor Cell, Mesenchymal or MSC or MSCs or Neuromuscular Blocking Agents or Neuromuscular Agents or Neuromuscular Blockade or Neuromuscular Depolarizing Agents or neuromuscular blockade or cisatracurium or alcuronium or atracurium or Boldine or curare or duranium or decamethonium or domoic acid or doxacurium or ethylcholine aziridinium or gallamine trimethoxide or grayanotoxin I or methoprene or Mivacurium or neosaxitoxin or pancuronium or pipecuronium or pyrantel or rapacuronium or Rocuronium or succinylcholine or tentoxin or tetanus toxin fragment C or toxiferines or tubocurarine or vecuronium bromide or vesamicol or inhaled vasodilators or nitric oxide or epoprostenol or iloprost or milrinone or prostacyclin].

In addition, we reviewed a large number of references found in the retrieved articles and sought other literature, such as research and conference reports. The scope of this study was limited to RCTs conducted in humans. The included literature was thoroughly reviewed to comprehensively identify relevant studies and avoid omissions. Two reviewers worked independently, examining titles and abstracts, summarizing search results, and applying the inclusion and exclusion criteria. The Cochrane Handbook^[18]^ was used to assess the risk of bias and the quality of the included trials. In cases of disagreement between the 2 reviewers, a third author made a significant decision.

### 2.3. Study selection

We followed the PICOS (population, interventions, comparison outcomes, study designs) when defining the eligibility criteria.

The following conditions were used: (a) RCT. (b) The diagnosis of ARDS conforms to the relevant diagnostic criteria for ARDS as outlined in “The American-European Consensus Conference on ARDS: Definitions, mechanisms, relevant outcomes, and clinical trial coordination” or “Acute respiratory distress syndrome: the Berlin definition.” There were no restrictions based on sex, age, disease course, or treatment duration. (c) The treatment group will receive pulmonary vasodilators, neuromuscular blocking agents, and stem cells. The control group will receive placebo and/or standard treatment (including oxygen therapy, bronchodilators, mucus clearance techniques, anti-infective agents, anti-inflammatory drugs, and nutritional support). (d) Outcomes: 28-day mortality, 90-day mortality, ventilator-free days, and oxygenation. (e) exclusion criteria.

Self-controlled trials, studies without a control group, interventions in the treatment and control groups that did not adhere to standard therapy, studies lacking full text, duplicate publications, and similar literature were excluded.

### 2.4. Literature screening

Literature inclusion and screening were conducted using Endnote software, initially through a combination of subject terms and free words to preliminarily retrieve relevant literature from various journal databases. Subsequently, the “remove duplicate titles” function was used to eliminate duplicate literature between databases. Further steps involved reviewing abstracts and full texts to exclude studies that did not meet the inclusion criteria. Two researchers independently completed the inclusion process, and the results were compared. Any discrepancies were resolved through group discussion or by consulting a third researcher. Data extraction was performed using a literature data extraction table created using Excel 2007.

### 2.5. Statistical analysis

We used Stata 17.0, to analyze the continuous variables extracted for NMA and generated results with 95% CI and standardized mean differences (SMD) or odds ratios (OR) with 95% CI. We employed a mixed model to assess statistical heterogeneity, with the criteria being *I*^2^ < 50% and *P* > .01. If these criteria were not met, a random effects model was used. Publication bias and small sample defects were evaluated using funnel plots. Each result was ranked based on surface under the cumulative ranking curve (SUCRA) values, with higher SUCRA values indicating better treatment effects. A matrix was established to compare all interventions, and the significance of the differences in SUCRA between each pair of interventions was examined. The consistency of these relationships was evaluated to enhance the stability of the results, with the statistical significance level set at *P* < .05.

## 3. Results

### 3.1. Literature search and included studies

**Figure 1.**
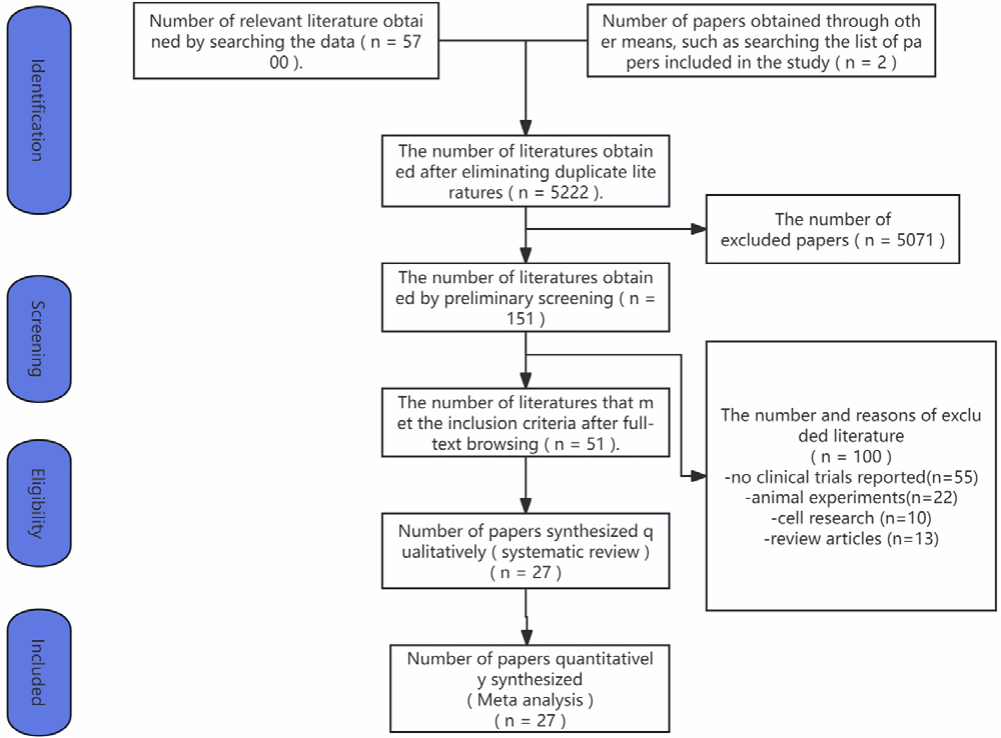
PRISMA flow diagram for search and selection of eligible studies included in the network meta-analysis. PRISMA = Preferred Reporting Items for Systematic Reviews and Meta-Analyses.

A flowchart of this study is presented in Figure 1. A total of 5700 records were identified through searches of databases and other sources, and after removing duplicate entries, 5222 records were evaluated. Following the screening of titles and abstracts, 5071 records were excluded, and after full-text reading, 124 records were excluded. Finally, we identified a total of 27 RCTs for inclusion, all of which were two-arm trials, involving a total of 3492 patients, with 1755 patients in the experimental group and 1737 patients in the control group. Detailed information is provided in Table 1.

**Table 1:** The information about the included studies.

References	Year	Sample size: I/C	Intervention	Ways	Dosage	Duration	Control	Result
Jean-Louis Vincent et al^[19]^	2001	70/32	Inhaled pulmonary vasodilators	Intravenous infusion	1.8 mg/kg/h	7 d	Placebo	①
Michael E. Bowdish et al^[20]^	2023	112/110	Mesenchymal stem cells	Intravenous infusion	2 × 10^6^ cells/kg	5 d	Placebo	①
Kazuya Ichikado et al^[21]^	2023	20/10	Mesenchymal stem cells	Intravenous infusion	9.0 × 10^8^ cells	/	Placebo	①②③
Herwig Gerlach et al^[22]^	2003	20/20	Inhaled pulmonary vasodilators	Inhalation	10 ppm	5 d	Placebo	②
Surabhi Chandra et al^[23]^	2020	29/30	Neuromuscular blocking agents	Intravenous infusion	1 μg/kg/min	48 h	Placebo	①
Lung M et al^[24]^	2019	501/505	Neuromuscular blocking agents	Intravenous infusion	37.5 mg/h	48 h	Placebo	①②③
Marc Gainnier et al^[25]^	2004	28/28	Neuromuscular blocking agents	Intravenous infusion	5.5 g/kg/min	48 h	Placebo	①③④
Christophe Guervilly et al^[26]^	2016	13/11	Neuromuscular blocking agents	Intravenous infusion	37.5 mg/h	48 h	Placebo	③
Cécile Pochon et al^[27]^	2023	15/15	Mesenchymal stem cells	Intravenous infusion	0.5 × 10^6^ cells/kg	6 d	Placebo	②③④
Eric troncy et al^[28]^	1998	15/15	Inhaled pulmonary vasodilators	Inhalation	2.5–40 ppm	2 d	Placebo	①
Schreiber MD et al^[29]^	2003	105/102	Inhaled pulmonary vasodilators	Inhalation	5 ppm	7 d	Placebo	②
Carlo Dani et al^[30]^	2006	20/20	Inhaled pulmonary Vasodilators	Inhalation	6 ppm	7 d	Placebo	②
Helene A. Haeberle et al^[31]^	2021	72/72	Inhaled pulmonary vasodilators	Inhalation	/	5 d	Placebo	②④
RW Taylor et al^[32]^	2004	192/193	Inhaled pulmonary vasodilators	Inhalation	5 ppm	28 d	Placebo	①
RA. Bronicki et al^[33]^	2015	26/29	Inhaled pulmonary vasodilators	Inhalation	5 ppm	28 d	Placebo	①
Laurent Papazian et al^[34]^	2010	178/162	Neuromuscular blocking agents	Intravenous infusion	37.5 mg/h	48 h	Placebo	①②③
Jean-Marie Forel MD et al^[35]^	2006	18/18	Neuromuscular blocking agents	Intravenous infusion	5.5 g/kg/min	48 h	Placebo	①③
H. James Ford et al^[36]^	2020	10/4	Inhaled pulmonary vasodilators	Inhalation	0.6 mg/mL	7 d	Placebo	①③
G. Bellingan et al^[37]^	2022	20/10	Mesenchymal stem cells	Intravenous infusion	9 × 10^8^ cells	96 h	Placebo	①②④
Najmeh Kaffash Farkhad et al^[38]^	2022	10/10	Mesenchymal stem cells	Intravenous infusion	1 × 10^6^ cells/kg	5 d	Placebo	①
Guoping Zheng et al^[39]^	2014	6/6	Mesenchymal stem cells	Intravenous infusion	1 × 10^6^ cells/kg	/	Placebo	③
Antoine Monsel et al^[40]^	2022	21/24	Mesenchymal stem cells	Intravenous infusion	1 × 10^6^ cells/kg	5 d	Placebo	①③④
Michael A Matthay et al^[41]^	2019	40/20	Mesenchymal stem cells	Intravenous infusion	1 × 10^6^ cells/kg	1 d	Placebo	①③
Giacomo Lanzoni et al^[42]^	2020	12/12	Mesenchymal stem cells	Intravenous infusion	100 × 10^6^ cells/kg	3 d	Placebo	①
Shahla Siddiqui et al^[43]^	2013	34/33	Inhaled pulmonary vasodilators	Inhalation	/	30 min	Placebo	④
G Lyu et al^[44]^	2014	48/48	Neuromuscular blocking agents	Intravenous infusion	0.05 mg/kg/h	48 h	Placebo	①
Dellinger R et al^[45]^	1998	120/57	Inhaled pulmonary vasodilators	Inhalation	1.25, 5, 20, 40, 80 ppm	28 d	Placebo	①④

Result: ① 28-day mortality, ② 90-day mortality, ③ ventilator-free days, ④ oxygenation improvement.

I/C = intervention/control; SOC = standard of care.

### 3.2. Synthesis of results

#### 3.2.1. 28 day mortality

**Figure 2.**
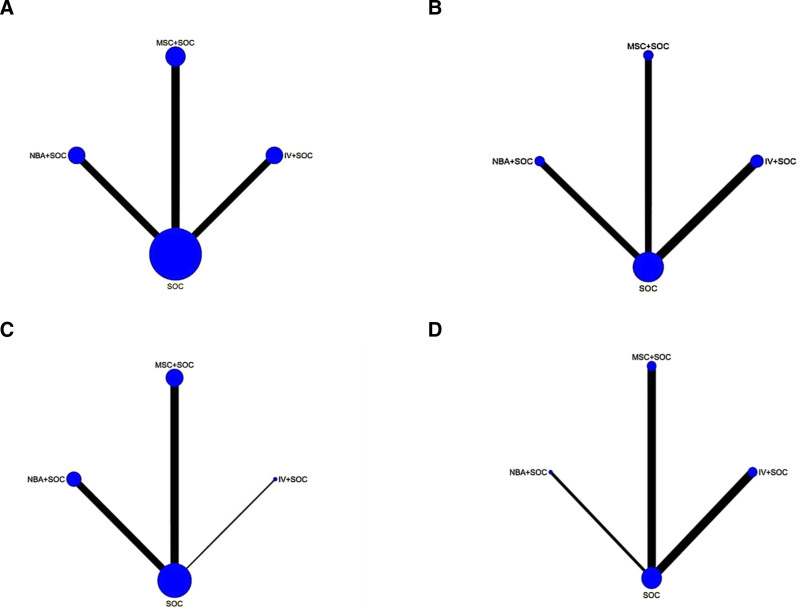
Network map for interventions with standard of care in outcome measures. (A) 28-day mortality; (B) 90-day mortality; (C) ventilator-free days; (D) oxygenation improvement. NBA + SOC: neuromuscular blocking agents + standard of care. MSC + SOC: mesenchymal stem cells + standard of care. IV + SOC: inhaled pulmonary vasodilators + standard of care. IV = inhaled pulmonary vasodilators, SOC = standard of care.

**Figure 3.**
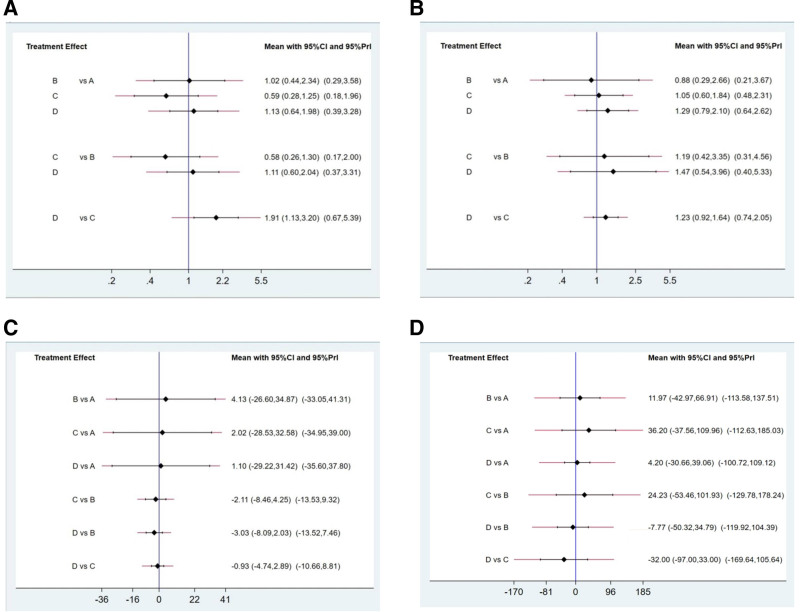
Forest plot ecacy of interventions with standard of care in outcome measures. A: 28-day mortality: (A) inhaled pulmonary vasodilators; (B) mesenchymal stem cells; (C) neuromuscular blocking agents; (D) standard of care. B: 90-day mortality: (A) inhaled pulmonary vasodilators; (B) mesenchymal stem cells; (C) neuromuscular blocking agents; (D) standard of care. C: Ventilator-free days: (A) inhaled pulmonary vasodilators; (B) mesenchymal stem cells; (C) neuromuscular blocking agents; (D) standard of care. D: Oxygenation improvement: (A) inhaled pulmonary vasodilators; (B) mesenchymal stem cells; (C) neuromuscular blocking agents; (D) standard of care.

The network estimate for the 28-day in-hospital mortality was based on 21 RCTs^[19–21,23–25,28,32–34,36–38,40–42,44,45]^ involving 2789 patients. Compared with standard treatment, neuromuscular blockers were associated with a reduced in-hospital mortality rate (OR 0.52, 95% CI 0.31–0.88), while IV and MSC were not significantly associated with reduced in-hospital mortality (OR 0.89, 95% CI 0.50–1.55; OR 0.90, 95% CI 0.49–1.66). There was no statistically significant difference in the association with reduced hospital mortality between MSC and IV and neuromuscular blockers (OR 0.98, 95% CI 0.43–2.25; OR 0.58, 95% CI 0.26–1.30). When comparing IV with neuromuscular blockers, there was no significant association with reduced hospital mortality (OR 0.59, 95% CI 0.28–1.25) (Table 2A). The network map is shown in Figure 2A. The forest plot ecacy of interventions is shown in Figure 3A.

The SUCRA rankings for reducing the 28-day mortality rate by intervention were neuromuscular blockers (93.5%), IV (42.4%), MSC (40.2%), and standard treatment (23.9%).

**Table 2:** Pairwise comparison matrix of interventions to outcomes (shown as mean difference and 95% confidence intervals).

A:			
(C)	1.69 (0.80,3.57)	1.72 (0.77,3.85)	1.91 (1.13,3.20)
0.59 (0.28,1.25)	(A)	1.02 (0.44,2.34)	1.13 (0.64,1.98)
0.58 (0.26,1.30)	0.98 (0.43,2.25)	(B)	1.11 (0.60,2.04)
0.52 (0.31,0.88)	0.89 (0.50,1.55)	0.90 (0.49,1.66)	(D)
B:			
(B)	1.14 (0.38,3.43)	1.27 (0.45,3.63)	1.47 (0.54,3.96)
0.88 (0.29,2.66)	(A)	1.12 (0.62,2.01)	1.29 (0.79,2.10)
0.79 (0.28,2.24)	0.89 (0.50,1.60)	(C)	1.15 (0.82,1.61)
0.68 (0.25,1.84)	0.77 (0.48,1.26)	0.87 (0.62,1.21)	(D)
C:			
(B)	‐2.11 (‐8.46,4.25)	‐4.13 (‐34.87,26.60)	‐3.03 (‐8.09,2.03)
2.11 (‐4.25,8.46)	(C)	‐2.02 (‐32.58,28.53)	‐0.93 (‐4.74,2.89)
4.13 (‐26.60,34.87)	2.02 (‐28.53,32.58)	(A)	1.10 (‐29.22,31.42)
3.03 (‐2.03,8.09)	0.93 (‐2.89,4.74)	‐1.10 (‐31.42,29.22)	(D)
D:			
(C)	‐24.23 (‐101.93,53.46)	‐32.00 (‐97.00,33.00)	‐36.20 (‐109.96,37.56)
24.23 (-53.46,101.93)	(B)	‐7.77 (‐50.32,34.79)	‐11.97 (‐66.91,42.97)
32.00 (‐33.00,97.00)	7.77 (-34.79,50.32)	(D)	‐4.20 (‐39.06,30.66)
36.20 (‐37.56,109.96)	11.97 (‐42.97,66.91)	4.20 (‐30.66,39.06)	(A)

A: 28-day mortality; B: 90-day mortality; C: ventilator-free days; D: oxygenation improvement.

(A) Inhaled pulmonary vasodilators; (B) mesenchymal stem cells; (C) neuromuscular blocking agents; (D) standard of care.

#### 3.2.2. 90 day mortality

The network estimate for the 90-day mortality rate was based on 9 RCTs^[21,22,24,27,29–31,34,37]^ involving 1862 patients. Compared to standard treatment, IV, MSC, and neuromuscular blockers were not significantly associated with reduced in-hospital mortality (OR 0.77, 95% CI 0.48–1.26; OR 0.68, 95% CI 0.25–1.84; OR 0.81, 95% CI 0.61–1.08). There was no statistically significant difference in the association with reduced hospital mortality between IV, MSC, and neuromuscular blockers when compared to each other (OR 0.95, 95% CI 0.54–1.67; OR 0.84, 95% CI 0.30–2.36). When comparing IV with neuromuscular blockers, there was no significant association with reduced hospital mortality (OR 0.88, 95% CI 0.29–2.66) (Table 2B).The network map is shown in Figure 2B.The forest plot ecacy of interventions is shown in Figure 3B.

According to the SUCRA rankings, MSCs are the most effective intervention for reducing the 90-day mortality rate is MSC. The ranking order from most effective to least effective is mesenchymal stem cells (67.6%), IV (64.5%), neuromuscular blockers (48.3%), and standard treatment (19.6%).

#### 3.2.3. Ventilator-free days

The network estimate for the number of ventilator-free days is based on 14 RCTs^[21,24–27,34–36,39–41]^ involving 1682 patients. Compared with standard treatment, IV, MSC, and neuromuscular blockers were not significantly associated with a reduction in ventilator-free days (SMD ‐1.10, 95% CI ‐31.42 to 29.22; SMD 3.03, 95% CI ‐2.03 to 8.09; SMD 0.93, 95% CI ‐2.89 to 4.74). There was no statistically significant difference in the association with ventilator-free days between IV, MSC, and neuromuscular blockers when compared to each other (SMD 4.13, 95% CI ‐26.60 to 34.87; SMD 2.02, 95% CI ‐28.53 to 32.58). When comparing neuromuscular blockers to MSC, there was no significant association between an increase in ventilator-free days (SMD 2.11, 95% CI ‐4.25 to 8.46) (Table 2C). The network map is shown in Figure 2C. The forest plot ecacy of interventions is shown in Figure 3C.

According to the SUCRA rankings, the ranking order of interventions for the effect on ventilator-free days is MSC (74.1%), neuromuscular blockers (49.8%), IV (43.9%), and standard treatment (32.2%).

#### 3.2.4. Oxygenation improvement

The network estimate for improvement in oxygenation, defined as the change in PaO_2_/FiO_2_ compared to baseline during treatment, was based on 8 RCTs^[25,27,31,37,40,43,45]^ involving 548 patients. Compared to standard treatment, IV, MSC, and neuromuscular blockers showed no statistically significant difference in improving oxygenation (SMD ‐4.20, 95% CI ‐39.06 to 30.66; SMD 7.77, 95% CI ‐34.79 to 50.32; SMD 32.00, 95% CI ‐33.00 to 97.00). When comparing IV to MSC and neuromuscular blockers, there was no significant association with improved oxygenation (SMD 11.97, 95% CI ‐42.97 to 66.91; SMD 36.20, 95% CI ‐37.56 to 109.96). When comparing MSC to neuromuscular blockers, the results regarding improvement in oxygenation were not significant (SMD 24.23, 95% CI ‐53.46 to 101.93) (Table 2D). The network map is shown in Figure 2D. The forest plot ecacy of interventions is shown in Figure 3D.

According to the SUCRA rankings, the ranking order of interventions for improving oxygenation is neuromuscular blockers (80.1%), MSC (52.1%), standard treatment (36.7%), and IV (31.1%).

#### 3.2.5. Quality assessment

**Figure 4.**
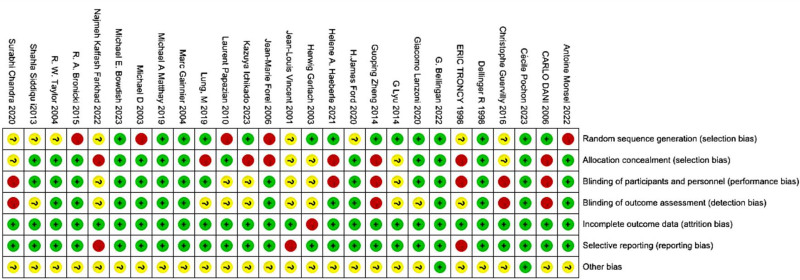
Risk of bias graph.

Fourteen (51.8%) randomized clinical trials showed a low risk of bias due to random number tables or random assignment using a computer number generator. Regarding assignment concealment, 14 (51.8%) trials had a lower risk because they used opaque envelopes or a central randomization system. Fifteen (55.5%) trials used blind methods for participants and staff, and 14 (51.8%) trials did not use blind methods for the evaluation of results. In all randomized clinical studies, the likelihood of selective reporting bias and incomplete outcome data is minimal. Other biases were not known in all included trials. As a result, the overall quality of the merged items is very high. Figure 4 shows the bias risk graph for the selected studies.

#### 3.2.6. Small sample effect detection

**Figure 5.**
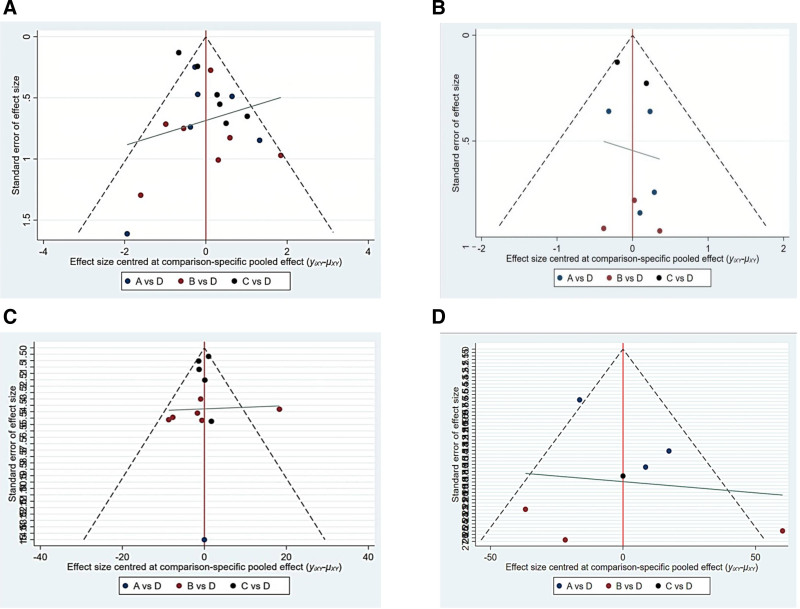
Comparison-adjusted funnel plots. (A) 28-day mortality; (B) 90-day mortality; (C) ventilator-free days; (D) oxygenation.

Comparison-adjusted funnel plots were created for the included studies (Fig. 5). Points of the same color in the funnel diagram symbolize pairwise comparisons in the original study, and points of different colors represent various pairwise direct comparisons. Symmetrical funnel plots (Fig. 5) indicated possible small-sample effects or little chance of publication bias for the 3 outcome measures between treatment measures.

#### 3.2.7. Adverse event

Among the 27 studies included in the network meta-analysis, 14 mentioned the occurrence of adverse events, among which sepsis, pneumothorax, thrombotic events, and cardiovascular disease were the most common, and the detailed adverse events were shown in Table 3.

**Table 3:** Adverse events included in the and page range literature.

Literature	Adverse event
Michael E. Bowdish et al^[20]^	Arrhythmia (ventricular, supraventricular), respiratory deterioration, hepatosis, sepsis, multisystem organ failure, myocardial infarction, hydrothorax, phrenoplegia, renal insufficiency, acute renal insufficiency, thromboembolic even, vasodilation state, pneumothorax, tumor
Kazuya Ichikado et al^[21]^	Arrhythmia, atrial fibrillation, increased sputum, abnormal hepatic function, pyrexia, increased blood pressure, increased pancreatic enzymes, and chills
Surabhi Chandra et al^[23]^	Hypotension
Lung M et al^[24]^	ICU acquired weakness, serious adverse cardiovascular events, ear barotrauma, pneumothorax
Christophe Guervilly et al^[26]^	Pneumothorax
Cécile Pochon et al^[27]^	Thromboembolic
Schreiber MD et al^[29]^	Intraventricular haemorrhage (IVH), periventricular leukomalacia (PVL), retinopathy of prematurity, necrotizing enterocolitis, sepsis
Helene A. Haeberle et al^[31]^	ICU acquired weakness, bleeding, VTE, need kidney replacement therapy, gastrointestinal complications, neurological complications, cardiovascular complication
RW Taylor et al^[32]^	Respiratory adverse events, creatinine elevation, pulmonary leakage syndrome (pneumothorax, emphysema, pneumopericardium)
Laurent Papazian et al^[35]^	Barotrauma, pneumothorax, MRC score
H. James Ford et al^[36]^	Blood and lymphatic system disorders, cardiac disorders, gastrointestinal disorders, general disorders, immune system disorders, injury, poisoning and procedural complications, nervous system disorders, renal and urinary disorders, respiratory, thoracic and mediastinal disorders, vascular disorders
G. Bellingan et al^[37]^	Sustained hypoxemia or hypotension and cardiac arrhythmia
Guoping Zheng et al^[39]^	Diarrhoea, multiple organ failure, sepsis
Antoine Monsel et al^[40]^	Diarrhoea

## 4. Discussion

This network meta-analysis provides valuable information for evaluating the impact of intensive care unit mortality, ventilator-free days, and oxygenation in patients with ARDS. Although there were no significant differences in 90-day mortality, ventilator-free days, or oxygenation improvement, neuromuscular blockers showed some advantages in reducing 28-day mortality compared to standard treatment. As ARDS is a life-threatening critical illness, its treatment is crucial, making this research result important for clinical practice. Current guidelines only recommend the use of corticosteroids to treat ARDS, with no clear guidance on the use of other medications. Therefore, these research findings offer valuable supplementary information; however, further discussion and research are still needed to determine the optimal treatment strategies and medication choices to enhance treatment outcomes and survival rates for patients with ARDS.

### 4.1. Neuromuscular blockers for ARDS

In the early stages of ARDS, neuromuscular blockers have been found to reduce ventilator-induced lung injury, decrease oxygen consumption, and exert anti-inflammatory effects. In ARDS, the use of lung-protective ventilation strategies can reduce the risk of ventilator-induced lung injury and improve survival rates.^[46,47]^ Neuromuscular blockers can control tidal volume and PEEP throughout the airway, reduce patient interaction with the ventilator, and minimize the risk of barotrauma, volumetric trauma, and atelectrauma.^[48]^ Additionally, research^[49]^ has shown that muscle paralysis can lead to decreased cardiac output and whole-body oxygen consumption. Neuromuscular blockers reduce the respiratory work of patients, which in turn reduces the oxygen consumption by the respiratory muscles. Moreover, neuromuscular blockers have anti-inflammatory effects, as early use of muscle relaxants has been associated with decreased concentrations of lung and systemic pro-inflammatory markers (such as IL-1β, IL-6, and IL-8),^[35]^ possibly due to the direct action of cisatracurium.^[50]^

In our meta-analysis, the RCTs included mainly vecuronium and cisatracurium as neuromuscular blockers. We found that early use of neuromuscular blockers can reduce 28-day mortality but has no significant impact on 90-day mortality, ventilator-free days, or oxygenation improvement. This aligns with the discussion in a 2021 review^[51]^ of ARDS, which suggested that neuromuscular blockers may have therapeutic value for carefully selected severe cases of ARDS, severe asynchrony with the ventilator, and refractory hypoxemia, especially those with a PaO_2_/FiO_2_ ratio <100 mm Hg. Similarly, the latest official clinical practice guidelines from the American Thoracic Society also recommend the use of neuromuscular blockers in early (within 48 hours of ARDS onset) and severe (PaO_2_/FiO_2_ ratio < 100 mm Hg) ARDS cases, limiting their use within 48 hours and emphasizing deeper sedation and analgesia when administering neuromuscular blockers.

### 4.2. Mesenchymal stem cells for ARDS

Our team included various sources of stem cells, including multipotent stromal cells (invimestrocel), Wharton jelly mesenchymal stromal cells, and allogeneic adipose-derived stem cells. According to the treatment time of MSC compared with the other 2 therapeutic measures, the treatment cycle of MSC is short and the intravenous injection is relatively simple, but its disadvantages are also prominent. During this period, the preparation of MSC is more complicated than that of other drugs. For example, the acute onset of graft-versus-host disease has a good curative effect, but for ARDS, the rapidness of the treatment time of MSC may be limited by the availability of stem cells, which needs to be further verified by clinical trials. Regarding the mechanism of action of MSC in treating ARDS, MSC, and their extracellular vesicles exert potent antimicrobial effects through direct and indirect mechanisms.^[52,53]^ Second, MSC can restore the permeability of the alveolar–capillary barrier, stabilizing lung endothelial barrier function through the secretion of hepatocyte growth factor^[54]^ in extracellular vesicles. Third, MSC can modulate immune responses and alleviate lung injuries. As a promising cell-based candidate therapy, MSC and their derived extracellular vesicles have the potential to modulate or suppress the innate and adaptive immune systems in various inflammatory diseases,^[55]^ including ARDS. Human MSC can enhance phagocytic capacity and promote an anti-inflammatory phenotype in human macrophages stimulated by lipopolysaccharide or ARDS bronchoalveolar lavage fluid through extracellular vesicle-mediated mitochondrial transfer.^[56]^ IL-27 has been identified as a pro-inflammatory factor in the pathophysiology of sepsis, with elevated levels of IL-27 in bronchoalveolar lavage fluid and serum of ALI/ARDS patients correlated with disease severity.^[57]^ Inhibition of IL-27 may present a promising therapeutic approach for ALI/ARDS patients.^[58]^ Extracellular vesicles from adipose-derived MSCs can alleviate sepsis-induced lung injury in mice by suppressing IL-27 secretion from macrophages.^[59]^ Fourth, substances carried by extracellular vesicles from MSC can inhibit cell death or regulate autophagy to improve ARDS. A study showed that EVs from adipose-derived stem cells specifically deliver miR-125b-5p^[60]^ to alleviate inflammation-induced pulmonary microvascular endothelial cell ferroptosis in sepsis-induced ALI by modulating Keap1/Nrf2/GPX4 expression, thereby improving acute lung injury. Another study demonstrated that EVs derived from bone marrow MSC^[61]^ can inhibit autophagic stress in alveolar macrophages by delivering miR-384-5p that directly binds to Beclin-1, thereby alleviating lipopolysaccharide-induced acute lung injury.

Although our meta-analysis showed no significant impact of MSC on in-hospital mortality, days without a ventilator, and oxygenation improvement in ARDS patients, MSC were superior to the other 2 types of medications in terms of 90-day mortality and days without a ventilator. In terms of safety, Matthay^[41]^ did not report any hemodynamic or respiratory adverse events related to MSC infusion in patients with moderate to severe ARDS.

### 4.3. Inhaled pulmonary vasodilator for ARDS

ARDS-induced pulmonary vascular changes, including thromboembolism and endothelial cell injury, can reduce pulmonary blood flow, leading to hypoxic vasoconstriction, deterioration of ventilation-perfusion matching, and increased dead-space ventilation.^[62]^ Alveolar-vascular mismatch and dead space ventilation manifest as refractory hypoxemia. Inhaled pulmonary vasodilators (such as nitric oxide and prostacyclin) are sometimes used as therapeutic options for ARDS to improve oxygenation and reduce dead space.^[51]^ Inhaled nitric oxide^[63]^ diffuses from the alveoli into pulmonary vascular smooth muscle cells, leading to vasodilation by increasing cyclic guanosine monophosphate. Prostacyclin acts on G protein-coupled receptors in the pulmonary vascular system to increase cyclic adenosine monophosphate, ultimately causing relaxation of the vascular smooth muscles.^[64]^ Inhaled pulmonary vasodilators can selectively dilate pulmonary blood vessels in well-ventilated alveoli, improving oxygenation by redirecting more blood to healthy alveoli.^[65]^ Although no clear benefits were observed in terms of mortality and other outcomes, a large cohort study^[2]^ showed that 13% of patients with severe ARDS were treated with IV.

The main interventions for IV in the RCTs included in our study were nitric oxide, prostacyclin, and prostaglandin E1. Our research indicates that IV did not have a significant impact on in-hospital mortality, days without a ventilator, or oxygenation improvement in patients with ARDS. Regarding in-hospital mortality, although CARLO DANI^[30]^ reported a significant reduction in the incidence and mortality of bronchopulmonary dysplasia in premature infants with severe respiratory distress syndrome after inhaled nitric oxide therapy, the combined endpoint used in this study was the occurrence of bronchopulmonary dysplasia and death. Although there was a significant decrease in the incidence of bronchopulmonary dysplasia, there was no significant change in mortality. In terms of oxygenation improvement, Haeberle et al^[31]^ showed a significant increase in the oxygenation index in the experimental group compared to the control group before the 5th day, while Dellinger R’s^[45]^ indicated that the improvement in the oxygenation index in the experimental group and the control group did not differ significantly from the 4th day onwards. This is similar to the results of 2 recent meta-analyses on IV, which suggest that inhaled nitric oxide or prostacyclin can improve oxygenation in the short term, but do not provide long-term benefits. The long-term inhalation of nitric oxide may lead to worsened oxygenation. Additionally, there may be a risk of kidney failure with prolonged use of IV.^[66]^ Therefore, we do not recommend nitric oxide as a long-term treatment option for patients with ARDS.

## 5. Limitations

This meta-analysis had several limitations. First, we only searched 3 databases and did not search other databases, such as Web of Science; however, we reviewed the reference lists of the most recent 4 systematic reviews to supplement our literature inclusion. Among the 27 RCTs, 19 studies had fewer than 50 patients in each group (ranging from to 4–48), and each comparison in the network meta-analysis included only a small number of studies, thus providing insufficient evidence for the effects of the 3 drugs in treating ARDS, and needing more multicenter clinical randomized controlled studies with large sample size. Second, among the included literatures, only 2 were grouped according to the severity of ARDS, accounting for only 7.4% of the literatures, which had little impact on the results. Moreover, we could not obtain the original RCT data and could not make statistics on the severity of ARDS, so subgroup analysis could not be conducted according to the severity of ARDS. Furthermore, we did not restrict the age of the patients, and 4 of the studies focused on infants; therefore, we did not conduct subgroup analyses and did not exclude the influence of age on the results. Third, in each RCT, standard treatment may differ owing to variations in experimental conditions, such as differences in mechanical ventilation strategies, corticosteroid use, and varying levels of sedation, which may impact the results. Finally, the 3 drugs were administered in different ways: nitric oxide was inhaled as an aerosol, neuromuscular blockade and MSC were administered intravenously. Although their main action is in the lungs, the possibility of interference caused by differences in the route of administration cannot be ruled out. Additionally, the dosage and duration of drug administration could also have influenced our results, as each study adopted different standards in this regard. Therefore, we cannot draw conclusions about the best treatment drugs, including in this study, and more experimental research is needed to confirm our results.

## 6. Conclusion

Neuromuscular blockade, IV, MSC, and standard treatment did not show significant differences in 90-day mortality, ventilator-free days, and PaO_2_/FIO_2_ ratio compared to baseline. However, compared to standard treatment, neuromuscular blockade may reduce 28-day mortality. Nonetheless, neuromuscular blockade may only have therapeutic value in specific severe cases of ARDS, severe dyssynchrony with the ventilator, and refractory hypoxemia.

## Author contributions

**Conceptualization:** Qing-Kuo Liu, Hao Hao.

**Data curation:** Qing-Kuo Liu, Guo-Han Xiang, Wen-Li Liu, Jin-Yan Dong, Yu-Qi Wen.

**Formal analysis:** Guo-Han Xiang, Yu-Qi Wen.

**Funding acquisition:** Hao Hao.

**Investigation:** Guo-Han Xiang, Jin-Yan Dong.

**Methodology:** Qing-Kuo Liu, Hao Hao, Guo-Han Xiang, Yu-Qi Wen.

**Project administration:** Qing-Kuo Liu, Wen-Li Liu.

**Resources:** Qing-Kuo Liu, Guo-Han Xiang, Jin-Yan Dong.

**Software:** Qing-Kuo Liu, Guo-Han Xiang, Wen-Li Liu, Yu-Qi Wen.

**Supervision:** Guo-Han Xiang, Wen-Li Liu.

**Validation:** Hao Hao, Jin-Yan Dong.

**Visualization:** Qing-Kuo Liu, Hao Hao, Jin-Yan Dong.

**Writing – original draft:** Qing-Kuo Liu, Wen-Li Liu, Jin-Yan Dong.

**Writing – review & editing:** Hao Hao.

## References

[1] Ranieri VM Rubenfeld GD Thompson BT. Acute respiratory distress syndrome: the Berlin definition. JAMA. 2012;307:2526–33. 22797452 10.1001/jama.2012.5669

[2] Bellani G Laffey JG Pham T. Epidemiology, patterns of care, and mortality for patients with acute respiratory distress syndrome in intensive care units in 50 countries. JAMA. 2016;315:788–800. 26903337 10.1001/jama.2016.0291

[3] Hasan SS Capstick T Ahmed R. Mortality in COVID-19 patients with acute respiratory distress syndrome and corticosteroids use: a systematic review and meta-analysis. Expert Rev Respir Med. 2020;14:1149–63. 32734777 10.1080/17476348.2020.1804365 PMC7544968

[4] Meyer NJ Gattinoni L Calfee CS. Acute respiratory distress syndrome. Lancet. 2021;398:622–37. 34217425 10.1016/S0140-6736(21)00439-6 PMC8248927

[5] Grasselli G Calfee CS Camporota L. ESICM guidelines on acute respiratory distress syndrome: Definition, phenotyping and respiratory support strategies. Intensive Care Med. 2023;49:727–59. 37326646 10.1007/s00134-023-07050-7 PMC10354163

[6] Lamontagne F Agarwal A Rochwerg B. A living WHO guideline on drugs for COVID-19. BMJ. 2020;370:m3379. 32887691 10.1136/bmj.m3379

[7] Dequin PF Meziani F Quenot JP. Hydrocortisone in severe communityacquired pneumonia. N Engl J Med. 2023;388:1931–41. 36942789 10.1056/NEJMoa2215145

[8] Savoie-White FH Tremblay L Menier CA. The use of early neuromuscular blockage in acute respiratory distress syndrome: a systematic review and meta-analyses of randomized clinical trials. Heart Lung. 2023;57:186–97. 36242824 10.1016/j.hrtlng.2022.10.001

[9] Tarazan N Alshehri M Sharif S. Neuromuscular blocking agents in acute respiratory distress syndrome: updated systematic review and meta-analysis of randomized trials. Intensive Care Med Exp. 2020;8:61. 33095344 10.1186/s40635-020-00348-6 PMC7582438

[10] Torbic H Krishnan S Harnegie MP Duggal A. Neuromuscular blocking agents for ARDS: a systematic review and meta-analysis. Respir Care. 2021;66:120–8. 32843506 10.4187/respcare.07849

[11] Wang F Li Y Wang B Li J Peng Z. The safety and efficacy of mesenchymal stromal cells in ARDS: a meta-analysis of randomized controlled trials. Crit Care. 2023;27:31. 36670442 10.1186/s13054-022-04287-4 PMC9857915

[12] Wang J Luo F Suo Y. Safety, efficacy and biomarkers analysis of mesenchymal stromal cells therapy in ARDS: A systematic review and meta-analysis based on phase I and II RCTs. Stem Cell Res Ther. 2022;13:275. 35752865 10.1186/s13287-022-02956-3 PMC9233855

[13] Torbic H Saini A Harnegie MP Sadana D Duggal A. Inhaled prostacyclins for acute respiratory distress syndrome: a systematic review and meta-analysis. Crit Care Explor. 2023;5:e0931. 37303944 10.1097/CCE.0000000000000931 PMC10256381

[14] Karam O Gebistorf F Wetterslev J Afshari A. The effect of inhaled nitric oxide in acute respiratory distress syndrome in children and adults: a cochrane systematic review with trial sequential analysis. Anaesthesia. 2017;72:106–17. 27762438 10.1111/anae.13628

[15] Khokher W Malhas SE Beran A. Inhaled pulmonary vasodilators in COVID-19 infection: a systematic review and meta-analysis. J Intensive Care Med. 2022;37:1370–82. 35915994 10.1177/08850666221118271 PMC9346441

[16] Moher D Liberati A Tetzlaff J Altman DG; PRISMA Group. Preferred reporting items for systematic reviews and meta-analyses: the PRISMA statement. PLoS Med. 2009;6:e1000097. 19621072 10.1371/journal.pmed.1000097 PMC2707599

[17] Hutton B Salanti G Caldwell DM. The PRISMA extension statement for reporting of systematic reviews incorporating network meta-analyses of health care interventions: checklist and explanations. Ann Intern Med. 2015;162:777–84. 26030634 10.7326/M14-2385

[18] Higgins JP Altman DG Gøtzsche PC. The Cochrane collaboration’s tool for assessing risk of bias in randomised trials. BMJ. 2011;343:d5928. 22008217 10.1136/bmj.d5928 PMC3196245

[19] Vincent JL Brase R Santman F. A multi-centre, double-blind, placebo-controlled study of liposomal prostaglandin E1 (TLC C-53) in patients with acute respiratory distress syndrome. Intensive Care Med. 2001;27:1578–83. 11685297 10.1007/s001340101077

[20] Bowdish ME Barkauskas CE Overbey JR. A randomized trial of mesenchymal stromal cells for moderate to severe acute respiratory distress syndrome from COVID-19. Am J Respir Crit Care Med. 2023;207:261–70. 36099435 10.1164/rccm.202201-0157OC PMC9896641

[21] Ichikado K Kotani T Kondoh Y. Clinical efficacy and safety of multipotent adult progenitor cells (invimestrocel) for acute respiratory distress syndrome (ARDS) caused by pneumonia: a randomized, open-label, standard therapy-controlled, phase 2 multicenter study (ONE-BRIDGE). Stem Cell Res Ther. 2023;14:217. 37608287 10.1186/s13287-023-03451-z PMC10464414

[22] Gerlach H Keh D Semmerow A. Dose-response characteristics during long-term inhalation of nitric oxide in patients with severe acute respiratory distress syndrome: a prospective, randomized, controlled study. Am J Respir Crit Care Med. 2003;167:1008–15. 12663340 10.1164/rccm.2108121

[23] Chandra S Goel S Dawra R. Early neuromuscular blockade in children with pediatric acute respiratory distress syndrome. J Pediatr Intensive Care. 2020;9:201–6. 32685248 10.1055/s-0040-1708557 PMC7360384

[24] Moss M Huang DT Brower RG. Early neuromuscular blockade in the acute respiratory distress syndrome. N Engl J Med. 2019;380:1997–2008. 31112383 10.1056/NEJMoa1901686 PMC6741345

[25] Gainnier M Roch A Forel J-M. Effect of neuromuscular blocking agents on gas exchange in patients presenting with acute respiratory distress syndrome. Crit Care Med. 2004;32:113–9. 14707568 10.1097/01.CCM.0000104114.72614.BC

[26] Guervilly C Bisbal M Forel JM. Effects of neuromuscular blockers on transpulmonary pressures in moderate to severe acute respiratory distress syndrome. Intensive Care Med. 2017;43:408–18. 28013329 10.1007/s00134-016-4653-4

[27] Pochon C Laroye C Kimmoun A. Efficacy of Wharton jelly mesenchymal stromal cells infusions in moderate to severe SARS-Cov-2 related acute respiratory distress syndrome: a phase 2a double-blind randomized controlled trial. Front Med. 2023;10:1224865. 10.3389/fmed.2023.1224865 PMC10495568 37706025

[28] Troncy E Collet JP Shapiro S. Inhaled nitric oxide in acute respiratory distress syndrome: a pilot randomized controlled study. Am J Respir Crit Care Med. 1998;157:1483–8. 9603127 10.1164/ajrccm.157.5.9707090

[29] Schreiber MD Gin-Mestan K Marks JD Huo D Lee G Srisuparp P. Inhaled nitric oxide in premature infants with the respiratory distress syndrome. N Engl J Med. 2003;349:2099–107. 14645637 10.1056/NEJMoa031154

[30] Dani C Bertini G Pezzati M Filippi L Cecchi A Rubaltelli FF. Inhaled nitric oxide in very preterm infants with severe respiratory distress syndrome. Acta Paediatr. 2006;95:1116–23. 16938760 10.1080/08035250600702594

[31] Haeberle HA Calov S Martus P. Inhaled prostacyclin improves oxygenation in patients with COVID-19-induced acute respiratory distress syndrome. medRxiv. 2023;24:58. 10.1186/s12931-023-02346-0 PMC9938510 36805707

[32] Taylor RW Zimmerman JL Dellinger RP. Low-dose inhaled nitric oxide in patients with acute lung injury: a randomized controlled trial. JAMA. 2004;291:1603–9. 15069048 10.1001/jama.291.13.1603

[33] Bronicki RA Fortenberry J Schreiber M Checchia PA Anas NG. Multicenter randomized controlled trial of inhaled nitric oxide for pediatric acute respiratory distress syndrome. J Pediatr. 2015;166:365–9.e1. 25454942 10.1016/j.jpeds.2014.10.011

[34] Papazian L Forel JM Gacouin A. Neuromuscular blockers in early acute respiratory distress syndrome. N Engl J Med. 2010;363:1107–16. 20843245 10.1056/NEJMoa1005372

[35] Forel JM Roch A Marin V. Neuromuscular blocking agents decrease inflammatory response in patients presenting with acute respiratory distress syndrome. Crit Care Med. 2006;34:2749–57. 16932229 10.1097/01.CCM.0000239435.87433.0D

[36] Ford HJ Anderson WH Wendlandt B. Randomized, placebo-controlled trial of inhaled treprostinil for patients at risk for acute respiratory distress syndrome. Ann Am Thorac Soc. 2021;18:641–7. 33095030 10.1513/AnnalsATS.202004-374OC

[37] Bellingan G Jacono F Bannard-Smith J. Safety and efficacy of multipotent adult progenitor cells in acute respiratory distress syndrome (MUST-ARDS): a multicentre, randomised, double-blind, placebo-controlled phase 1/2 trial. Intensive Care Med. 2022;48:36–44. 34811567 10.1007/s00134-021-06570-4 PMC8608557

[38] Farkhad NK Sedaghat A Reihani H. Specific clinical and immunological changes following mesenchymal stem cell transplantation in COVID-19–induced acute respiratory distress syndrome patients: a phase-I clinical trial. Iran J Allergy Asthma Immunol. 2022;21:687–703. 36640060 10.18502/ijaai.v21i6.11530

[39] Zheng G Huang L Tong H. Treatment of acute respiratory distress syndrome with allogeneic adipose-derived mesenchymal stem cells: a randomized, placebo-controlled pilot study. Respir Res. 2014;15:1–10. 24708472 10.1186/1465-9921-15-39 PMC3994204

[40] Monsel A Hauw-Berlemont C Mebarki M. Treatment of COVID-19-associated ARDS with mesenchymal stromal cells: a multicenter randomized double-blind trial. Crit Care. 2022;26:48. 35189925 10.1186/s13054-022-03930-4 PMC8860258

[41] Matthay MA Calfee CS Zhuo H. Treatment with allogeneic mesenchymal stromal cells for moderate to severe acute respiratory distress syndrome (START study): a randomised phase 2a safety trial. Lancet Respir Med. 2019;7:154–62. 30455077 10.1016/S2213-2600(18)30418-1 PMC7597675

[42] Lanzoni G Linetsky E Correa D. Umbilical cord mesenchymal stem cells for COVID-19 acute respiratory distress syndrome: a double-blind, phase 1/2a, randomized controlled trial. Stem Cells Transl Med. 2021;10:660–73. 33400390 10.1002/sctm.20-0472 PMC8046040

[43] Siddiqui S Salahuddin N Zubair S Yousuf M Azam I Gilani AH. Use of inhaled PGE1 to improve diastolic dysfunction, LVEDP, pulmonary hypertension and hypoxia in ARDS—a randomised clinical trial. Open J Anesthesiol. 2013;03:109.

[44] Lyu G Wang X Jiang W Cai T Zhang Y. Clinical study of early use of neuromuscular blocking agents in patients with severe sepsis and acute respiratory distress syndrome. Zhonghua wei zhong bing ji jiu yi xue. 2014;26:325–9. 24809261 10.3760/cma.j.issn.2095-4352.2014.05.008

[45] Dellinger RP Zimmerman JL Taylor RW. Effects of inhaled nitric oxide in patients with acute respiratory distress syndrome: results of a randomized phase II trial. Crit Care Med. 1998;26:15–23. 9428538 10.1097/00003246-199801000-00011

[46] Brower RG Matthay MA Morris A. Ventilation with lower tidal volumes as compared with traditional tidal volumes for acute lung injury and the acute respiratory distress syndrome. N Engl J Med. 2000;342:1301–8. 10793162 10.1056/NEJM200005043421801

[47] Amato MB Meade MO Slutsky AS. Driving pressure and survival in the acute respiratory distress syndrome. N Engl J Med. 2015;372:747–55. 25693014 10.1056/NEJMsa1410639

[48] Tsolaki V Zakynthinos GE Papadonta ME. Neuromuscular blockade in the pre- and COVID-19 ARDS patients. J Pers Med. 2022;12:1538. 36143323 10.3390/jpm12091538 PMC9504585

[49] Marik PE Kaufman D. The effects of neuromuscular paralysis on systemic and splanchnic oxygen utilization in mechanically ventilated patients. Chest. 1996;109:1038–42. 8635328 10.1378/chest.109.4.1038

[50] Fanelli V Morita Y Cappello P. Neuromuscular blocking agent cisatracurium attenuates lung injury by inhibition of nicotinic acetylcholine receptor-α1. Anesthesiology. 2016;124:132–40. 26540149 10.1097/ALN.0000000000000907

[51] Qadir N Chang SY. Pharmacologic treatments for acute respiratory distress syndrome. Crit Care Clin. 2021;37:877–93. 34548139 10.1016/j.ccc.2021.05.009 PMC8449143

[52] Alcayaga-Miranda F Cuenca J Khoury M. antimicrobial activity of mesenchymal stem cells: current status and new perspectives of antimicrobial peptide-based therapies. Front Immunol. 2017;8:339. 28424688 10.3389/fimmu.2017.00339 PMC5371613

[53] Monsel A Zhu YG Gennai S. Therapeutic effects of human mesenchymal stem cell-derived microvesicles in severe pneumonia in mice. Am J Respir Crit Care Med. 2015;192:324–36. 26067592 10.1164/rccm.201410-1765OC PMC4584251

[54] Wang H Zheng R Chen Q Shao J Yu J Hu S. Mesenchymal stem cells microvesicles stabilize endothelial barrier function partly mediated by hepatocyte growth factor (HGF). Stem Cell Res Ther. 2017;8:211. 28969681 10.1186/s13287-017-0662-7 PMC5623961

[55] Buzas EI. The roles of extracellular vesicles in the immune system. Nat Rev Immunol. 2023;23:236–50. 35927511 10.1038/s41577-022-00763-8 PMC9361922

[56] Morrison TJ Jackson MV Cunningham EK. Mesenchymal stromal cells modulate macrophages in clinically relevant lung injury models by extracellular vesicle mitochondrial transfer. Am J Respir Crit Care Med. 2017;196:1275–86. 28598224 10.1164/rccm.201701-0170OC PMC5694830

[57] Fan J Zhang YC Zheng DF. IL-27 is elevated in sepsis with acute hepatic injury and promotes hepatic damage and inflammation in the CLP model. Cytokine. 2020;127:154936. 31786500 10.1016/j.cyto.2019.154936

[58] Xu F Liu Q Lin S Shen N Yin Y Cao J. IL-27 is elevated in acute lung injury and mediates inflammation. J Clin Immunol. 2013;33:1257–68. 23842867 10.1007/s10875-013-9923-0 PMC7102048

[59] Wang X Liu D Zhang X Yang L Xia Z Zhang Q. Exosomes from adipose-derived mesenchymal stem cells alleviate sepsis-induced lung injury in mice by inhibiting the secretion of IL-27 in macrophages. Cell Death Discov. 2022;8:18. 35013123 10.1038/s41420-021-00785-6 PMC8744023

[60] Shen K Wang X Wang Y. miR-125b-5p in adipose derived stem cells exosome alleviates pulmonary microvascular endothelial cells ferroptosis via Keap1/Nrf2/GPX4 in sepsis lung injury. Redox Biol. 2023;62:102655. 36913799 10.1016/j.redox.2023.102655 PMC10023991

[61] Liu X Gao C Wang Y Niu L Jiang S Pan S. BMSC-derived exosomes ameliorate LPS-induced acute lung injury by miR-384-5p-controlled alveolar macrophage autophagy. Oxid Med Cell Longev. 2021;2021:9973457. 34234888 10.1155/2021/9973457 PMC8216833

[62] Tomashefski JF Jr Davies P Boggis C Greene R Zapol WM Reid LM. The pulmonary vascular lesions of the adult respiratory distress syndrome. Am J Pathol. 1983;112:112–26. 6859225 PMC1916312

[63] Yu B Ichinose F Bloch DB Zapol WM. Inhaled nitric oxide. Br J Pharmacol. 2019;176:246–55. 30288739 10.1111/bph.14512 PMC6295404

[64] Del Pozo R Hernandez Gonzalez I Escribano-Subias P. The prostacyclin pathway in pulmonary arterial hypertension: a clinical review. Expert Rev Respir Med. 2017;11:491–503. 28399721 10.1080/17476348.2017.1317599

[65] Nasrullah A Virk S Shah A. Acute respiratory distress syndrome and the use of inhaled pulmonary vasodilators in the COVID-19 era: a narrative review. Life (Basel). 2022;12:1766. 36362921 10.3390/life12111766 PMC9695622

[66] Gebistorf F Karam O Wetterslev J Afshari A. Inhaled nitric oxide for acute respiratory distress syndrome (ARDS) in children and adults. Cochrane Database Syst Rev. 2016;2016. 10.1002/14651858.CD002787.pub3 PMC6464789 27347773

